# Arachidonic acid has a dominant effect to regulate lipogenic genes in 3T3-L1 adipocytes compared to omega-3 fatty acids

**DOI:** 10.3402/fnr.v59.25866

**Published:** 2015-03-20

**Authors:** Hitesh Vaidya, Sukhinder Kaur Cheema

**Affiliations:** Department of Biochemistry, Memorial University, St. John's, NL, Canada

**Keywords:** adipocytes, arachidonic acid, lipogenesis, omega-3 polyunsaturated fatty acids

## Abstract

**Background:**

The effects of long-chain n-3 and n-6 polyunsaturated fatty acids (PUFA) on the regulation of adipocytes metabolism are well known. These fatty acids are generally consumed together in our diets; however, the metabolic regulation of adipocytes in the presence of these fatty acids when given together is not known.

**Objective:**

To investigate the effects of n-3 PUFA and arachidonic acid (AA), an n-6 PUFA, on the regulation of adipogenic and lipogenic genes in mature 3T3-L1 adipocytes.

**Methods:**

3T3-L1 adipocytes were incubated in the presence or absence of 100 µM of eicosapentaenoic acid, EPA; docosahexaenoic acid, DHA; docosapentaenoic acid, DPA and AA, either alone or AA+n-3 PUFA; control cells received bovine serum albumin alone. The mRNA expression of adipogenic and lipogenic genes was measured. The fatty acid composition of adipocytes was analyzed using gas chromatography.

**Results:**

Individual n-3 PUFA or AA had no effect on the mRNA expression of peroxisome-proliferator-activated receptor-γ; however, AA+EPA and AA+DPA significantly increased (*P*<0.05) the expression compared to control cells (38 and 42%, respectively). AA and AA+EPA increased the mRNA expression of acetyl-CoA carboxylase 1 (*P*<0.05). AA treatment decreased the mRNA expression of stearoyl-CoA desaturase (SCD1) (*P*<0.01), while n-3 PUFA, except EPA, had no effect compared to control cells. AA+DHA and AA+DPA inhibited SCD1 gene expression (*P*<0.05) suggesting a dominant effect of AA. Fatty acids analysis of adipocytes revealed a higher accretion of AA compared to n-3 PUFA.

**Conclusions:**

Our findings reveal that AA has a dominant effect on the regulation of lipogenic genes in adipocytes.

Polyunsaturated fatty acids (PUFA) are generally considered to have beneficial health effects; however, there are opposing effects of omega (n)-3 and n-6 PUFA in regulating metabolic functions (1). Diets enriched in n-6 PUFA are suggested to be associated with obesity (2, 3), while diets enriched in n-3 PUFA protect against obesity (4, 5). The opposing effects of n-3 and n-6 PUFA are likely due to differential effects of these fatty acids to regulate adipogenesis, lipogenesis, and lipolytic pathways (3, 6, 7). Adipogenesis and lipogenesis involve triglyceride accumulation in adipocytes; however, triglyceride stores in mature adipocytes are in constant flux. Dietary status, especially dietary fats, plays an important role in regulating triglyceride homeostasis (8). Although individual effects of long-chain (LC) n-3 and n-6 PUFA on lipid and triglyceride metabolism in adipocytes are well studied (9–11), there are limited studies to show the effects of these fatty acids when given together (12). This is important as both LC n-3 and n-6 PUFA are generally consumed together in our diet. We hypothesized that a combination of specific LC n-3 PUFA and AA will have a differential effect on the regulation of adipogenic and lipogenic genes compared to the individual effects of LC-PUFA. It was further hypothesized that the differential effects will be due to a higher incorporation of specific fatty acids in mature adipocytes. Fully differentiated adipocytes were used as these represent mature non-insulin-resistant fat cells, which are metabolically active (13). Our findings reveal for the first time that arachidonic acid (AA) has a predominant effect on the regulation of lipogenic genes when given together in an equal combination with specific LC n-3 PUFA.

## Material and methods

### Treatment of 3T3-L1 cells with fatty acids

3T3-L1 pre-adipocytes were obtained from American Type Culture Collection (ATCC # CL-173, USA) and maintained in Dulbecco's Modified Eagle's Medium (DMEM) (Gibco, USA) containing 10% calf serum (Gibco, USA) in a 5% CO_2_, humidified environment at 37°C. Differentiation of confluent preadipocytes cultures (Day 0) was induced (14) and cells were differentiated over an 8-day period, with Day 8 representing fully differentiated mature adipocytes.

Pure fatty acids (Sigma Aldrich, USA) were complexed to fatty acid free bovine serum albumin (BSA) (MP Biochemical, USA). Briefly, 0.1 M stock solution of fatty acids in ethanol was diluted 1:100 into 2% (W/V) fatty acid free-BSA in DMEM at 60°C with shaking. Fully differentiated adipocytes were incubated in serum-free medium (DMEM+0.1% BSA) for overnight and incubated for 48 h in fresh DMEM containing 2% BSA in the presence or absence of 100 µM of n-3 PUFA (eicosapentaenoic acid, EPA; docosahexaenoic acid, DHA; docosapentaenoic acid, DPA) and AA (n-6 PUFA) individually, or an equal combination of 100 µM of AA+EPA, AA+DHA, AA+DPA in 1:1 ratio. Control cells received BSA alone. The selected concentration of fatty acids is within the physiological range for free fatty acids concentration for rodents and humans (15). Cells were washed with 1X phosphate buffered saline to either extract total RNA or to extract lipids.

### Total RNA isolation and measurement of gene expression

Total RNA was isolated from the cells using TriZol kit (Invitrogen, USA) according to the manufacture's instruction. RNA concentration and purity was measured using NanoDrop 2000 (Thermo Scientific, USA). Primers used for RT-PCR were designed using NCBI primer blast (www.ncbi.nlm.nih.gov/tools/primer-blast/), and obtained from IDT technologies (IA, USA). Primer sequences for peroxisome-proliferator-activated receptor (PPAR)-γ, adiponectin, acetyl-CoA carboxylase 1 (ACC1), stearoyl-CoA desaturase1 (SCD1), and β-actin are given in Table 1. To ensure optimal DNA polymerization efficiency and amplification specificity, amplicon length was set between 100 and 300 bp. Total RNA was treated with DNAse and first-strand cDNA was synthesized from 1 µg of total RNA using M-MLV Reverse Transcriptase (Promega, Canada) and random primers as per our previous publication (16). The cDNA templates were used for *in vitro* DNA amplification using specific primers and beta-actin simultaneously. Amplification was performed for 30 cycles under the following conditions: 94°C for 5 min for the first cycle and 1 min for subsequent cycles, 60°C for 1 min, and 72°C for 1 min. The total number of cycles for PCR reaction was chosen to remain within the exponential phase of the reaction. All PCR reactions were performed in triplicate and the products were separated by electrophoresis on a 2.0% agarose gel; no amplification products were detected in the absence of reverse transcriptase. The RT-PCR products were visualized using SYBR Safe DNA gel stain (Invitrogen) and analyzed using Chemi-Imager 4400, normalized to β-actin expression and expressed as percentage change.

**Table 1:** Sequence of primers used for reverse transcription-polymerase chain reaction

Primers	Forward	Reverse
Peroxisome-proliferator-activated receptor-γ	5′-GAGCTGACCCAATGGTTGCTG-3′	5′-GCTTCAATCGGATGGTTCTTC-3′
Adiponectin	5′-GCCCAGTCATGCCGAAGA-3′	5′-TCTCCAGCCCCACACACTGAAC-3′
Acetyl-CoA carboxylase 1	5′-GGACCACTGCATGGAATGTTA-3′	5′-TGAGTGACTGCCGAAACATCTC-3′
Stearoyl-CoA desaturase1	5′-CACCTGCCTCTTCGGGATTT-3′	5′-CTTTGACAGCCGGGTGTTTG-3′
β-actin	5′-ATGGTGGGAATGGGTCAGAAG-3′	5'CACGCAGCTCATTGTAGAAGG-3′

### Fatty acid analysis

Total lipids were extracted using the Folch extraction method (17) and fatty acid methyl esters were prepared and analyzed by gas chromatography as per our previous publication (18).

## Statistical analysis

The results were analyzed using one-way analysis of variance (ANOVA) followed by Dunnett's and Tukey's multiple tests to compare each treatment group with the control group. All experiments were performed in duplicate with *n*=3 in each group. A value of *P*<0.05 was considered to be significant. The results were expressed as mean±SD. The statistical analysis was carried out by using Graph Pad 5.0 software.

## Results

### Adipogenic and lipogenic genes are differentially regulated when 3T3-L1 cells are treated with individual or an equal combination of LC n-3 PUFA and AA

**Fig. 1:**
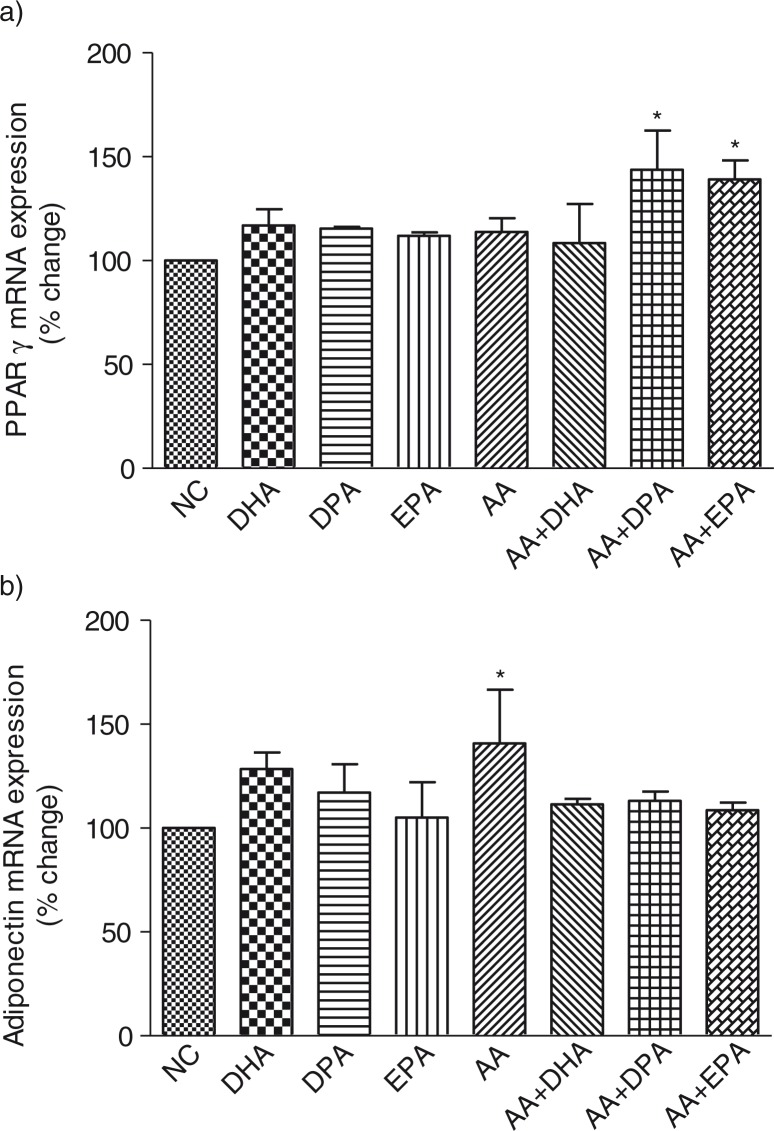
Effects of long-chain n-3 polyunsaturated fatty acids and arachidonic acid on the mRNA expression of peroxisome-proliferator-activated receptor (PPAR)-γ (a) and adiponectin (b) in fully differentiated 3T3-L1 adipocytes. Fully differentiated adipocytes were treated with either individual long-chain polyunsaturated fatty acids (LC-PUFA) or an equal combination of AA+LC n-3 PUFA as explained in the methods section. The mRNA expression was normalized to β-actin. Each bar is represented as mean±SD. Results were analyzed using one-way analysis of variance (ANOVA) and Dunnett's multiple tests was performed to check statistical significant effects. Asterisk (*) indicates *P<*0.05 significantly different compared to control cells. NC=normal control, DHA=docosahexaenoic acid, EPA=eicosapentaenoic acid, DPA=docosapentaenoic acid, AA=arachidonic acid, SD=standard deviation.

Treatment with individual LC n-3 PUFA and AA had no significant effect on the mRNA expression of PPAR-γ compared to the control cells; however, treatment with AA+EPA and AA+DPA significantly (*P*<0.05) increased the mRNA expression of PPAR-γ compared to the control cells (42.7 and 38.1%, respectively) (Fig. 1a). Only AA significantly (*P*<0.05) increased the mRNA expression of adiponectin compared to the control cells (Fig. 1b).

**Fig. 2:**
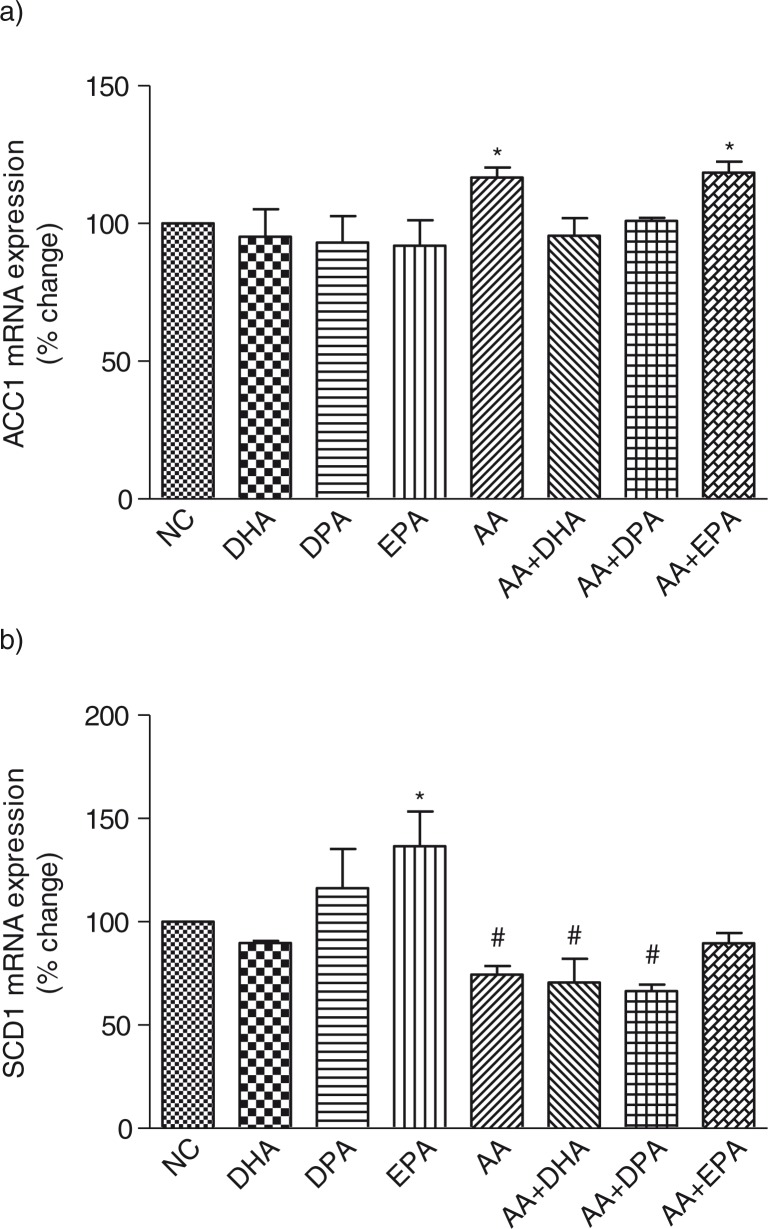
Effects of long-chain n-3 polyunsaturated fatty acids and AA on the mRNA expression of acetyl-CoA carboxylase 1 (ACC1) (a) and stearoyl-CoA desaturase 1 (SCD1) (b) in fully differentiated 3T3-L1 adipocytes. Fully differentiated adipocytes were treated with either individual long-chain polyunsaturated fatty acids (LC-PUFA) or an equal combination of AA+ LC n-3 PUFA as explained in the methods section. The mRNA expression was normalized to β-actin. Each bar is represented as mean±SD. Results were analyzed using one-way analysis of variance (ANOVA) and Dunnett's multiple tests was performed to check statistically significant effects. Asterisk (*) and the number (#) symbol indicates significantly different (*P*<0.05) compared to control cells. NC=normal control, DHA=docosahexaenoic acid, EPA=eicosapentaenoic acid, DPA=docosapentaenoic acid and AA=arachidonic acid, SD=standard deviation.

LC n-3 PUFA showed no effect on the mRNA expression of ACC1 (Fig. 2a); however, AA and AA+EPA treatment caused a significant (*P*<0.05) increase (17% increase) in the mRNA expression of ACC1 compared to the control cells (Fig. 2a). Treatment with DHA and DPA had no effect on the mRNA expression of SCD1 compared to the control cells, while EPA showed an increase (36%) and AA decreased the mRNA expression of SCD1 (25%) (Fig. 2b). On the contrary, treatment with AA+DHA and AA+DPA significantly (*P*<0.05) decreased the mRNA expression of SCD1 in mature 3T3-L1 adipocytes (29 and 33%, respectively) (Fig. 2b) compared to control cells. It was interesting to note that AA+EPA showed no significant effect on SCD1 mRNA expression.

### 3T3-L1 cells have a higher accretion of AA when treated with an equal combination of LC n-3 PUFA and AA

**Fig. 3:**
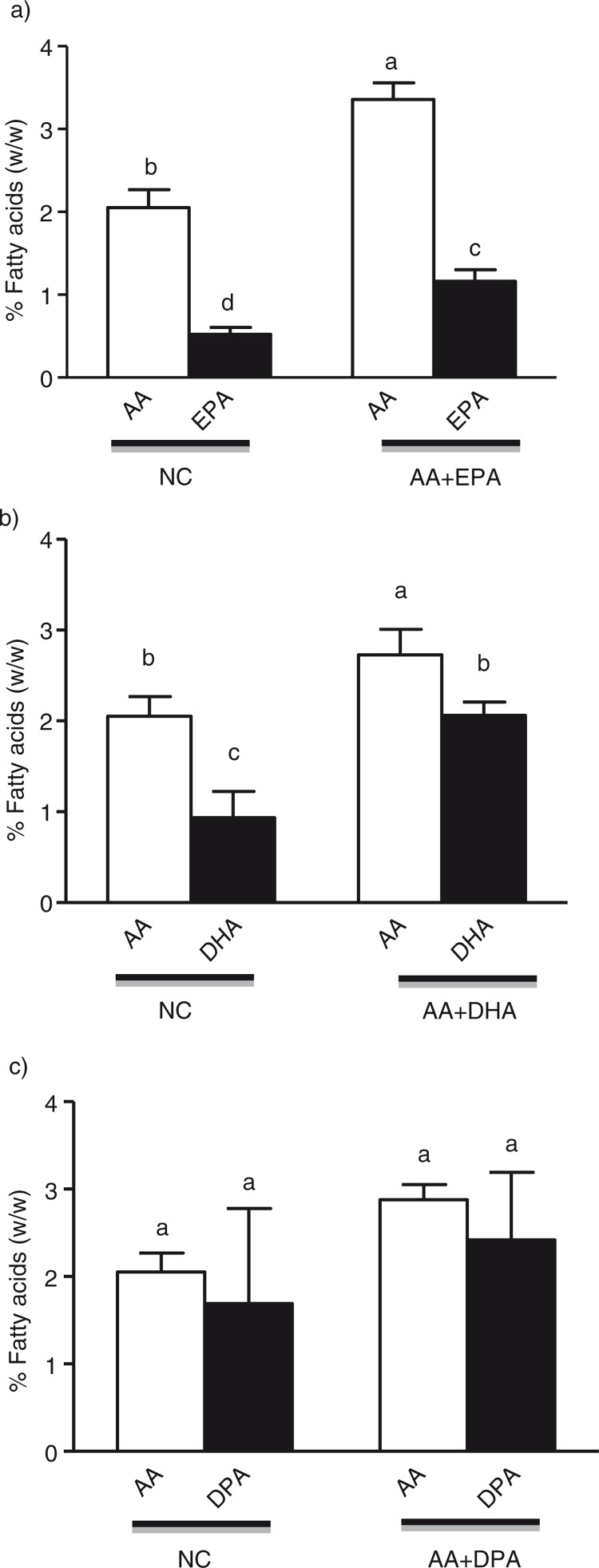
Effect of an equal combination of AA with specific long-chain n-3 polyunsaturated fatty acids on the fatty acid concentration of fully differentiated 3T3-L1 adipocytes. Cells were treated with AA+EPA (a), AA+DHA (b) and AA+DPA (c) in 1:1 ratio, and control cells (NC) received BSA alone as explained under the methods section. Total lipids were extracted and fatty acid analysis was performed. Each bar is represented as mean±SD. Results were analyzed using one-way analysis of variance (ANOVA) and Tukey's multiple tests was performed to check statistical significant effects. Different superscripts represent significant differences, *P*<0.05. AA=arachidonic acid, DHA=docosahexaenoic acid, EPA=eicosapentaenoic acid, DPA=docosapentaenoic acid, SD=standard deviation.

Treatment with an equal combination of specific LC n-3 PUFA and AA showed an increase in the specific fatty acids incorporated into mature adipocytes (Fig. 3a–c). An equal combination of AA+EPA treated cells showed significantly higher amounts of AA compared to the respective LC n-3 PUFA (Fig. 3a); however, treatment with AA+DHA showed higher incorporation of DHA compared to AA (Fig. 3b). This effect was not significant in AA+DPA-treated group (Fig. 3c). Treatment with AA+EPA showed an increase of 1.31% for AA and 0.64% for EPA in the adipocytes compared to normal control group. Treatment with AA+DHA showed an increase of 0.67% for AA and 1.13% for DHA in the adipocytes compared to normal control cells.

## Discussion

Studies have emphasized the proadipogenic properties of n-6 PUFA, and have provided evidence that rodents fed with diets high in n-6 PUFA have an increased fat mass (3). On the contrary, a handful of studies have shown beneficial effects of n-3 PUFA on obesity (5), suggesting opposing effects of n-3 and n-6 LC-PUFA on the regulation of adipocytes metabolism. We have investigated the effects of LC n-3 PUFA and AA, an n-6 PUFA, either individually or in equal combination, on adipogenic and lipogenic genes using fully differentiated mature 3T3-L1 adipocytes. Mature adipocytes were used as these are metabolically active and represent mature non-insulin resistant fat cells (13). In mature adipocytes, PPAR-γ is the central regulator of adipogenesis and plays a dominant role in fat tissue development. It also maintains adipocytes in the terminal differentiation stage (19). PPAR-γ agonists have been shown to decrease insulin resistance by increasing the capacity of fat cells to store lipids (20) and to augment adiponectin expression, which contributes to a lower risk of insulin resistance (21).

Treatment with individual LC n-3 PUFA and AA showed a trend toward an increase in the mRNA expression of PPAR-γ; however, the effect was not statistically significant. On the contrary, an equal combination of AA+DPA and AA+EPA significantly increased the mRNA expression of PPAR-γ. The increase in PPAR-γ mRNA expressions with individual DPA, EPA, and AA treatment was 15.4, 11.9, and 13.7%, respectively; however, treatment with AA+DPA and AA+EPA increased the mRNA expression of PPAR-γ by 42.7 and 38.1%, respectively, suggesting a synergistic effect of AA and LC n-3 PUFA on PPAR-γ gene expression. Adiponectin mRNA expression was significantly increased by AA treatment compared to control cells, while DPA and EPA had no effect. Oster et al. (10) has reported an increase in PPAR-γ and adiponectin mRNA expression after 24 h of treatment with DHA in differentiated adipocytes, with no effect of EPA on PPAR-γ or adiponectin gene expression.

Adipogenesis process involves lipid accumulation, which depends on the uptake of circulating fatty acids (22) and also *de novo* synthesis involving ACC1, a rate limiting enzyme in fatty acid biosynthesis. N-3 PUFA has been shown to decrease the expression of lipogenic genes in 3T3-L1 adipocytes (23); however, the effects of LC n-3 and n-6 PUFA when given together have not been well studied in differentiated adipocytes. Treatment with AA showed a small but significant increase in the mRNA expression of ACC1 (17%) in fully differentiated adipocytes compared to control cells. Peng et al. (24) found an increase in ACC1 gene expression after treatment with AA in hepatic cells compared to control group; however, we are the first to report the effects of AA on ACC1 mRNA expression in mature adipocytes. LC n-3 PUFA showed no effect on the mRNA expression of ACC1, which is contrary to the findings of Lee et al. (11) to show a decrease in ACC1 gene expression in differentiated adipocytes treated with EPA. These authors however used a much higher dose of EPA (300 µM). Treatment with AA+EPA also showed a small but significant increase in ACC1 gene expression compared to control cells (17% increase), which is similar to the increase found with AA treatment suggesting that the effect is due to AA. However, treatment with AA+DPA or AA+DHA had no effect on ACC1 gene expression, suggesting that DPA and DHA are protective against AA-induced increase in ACC1 gene expression. Inhibition of ACC1 is beneficial in obesity and insulin resistance (25); thus, a dominant effect of AA over LC n-3 PUFA to increase ACC1 gene expression would have detrimental effects.

Another enzyme that plays a central role in lipogenesis process is SCD1 that catalyzes the rate limiting step in the conversion of saturated to monounsaturated fatty acids (26). SCD1 is highly expressed in adipocytes (27); alterations in SCD1 regulation have been implicated in metabolic disorders such as obesity, diabetes, atherosclerosis, and inflammation (26). Treatment with DHA and DPA had no effect on SCD1 gene expression; however, EPA significantly increased the mRNA expression of SCD1 compared to control cells. Sessler et al. (28) has previously reported that treatment of mature adipocytes with EPA decreased SCD1 gene expression; however, these authors used a much higher dose of EPA (300 µM). Contrary to the effect of EPA, AA significantly decreased SCD1 mRNA expression compared to control cells. DHA showed a decrease in mRNA expression of SCD1 but this effect was not statistically significant; however, treatment with AA+DHA significantly inhibited SCD1 mRNA expression compared to control cells suggesting a synergistic and dominant effect of AA. Although DPA alone had no effect on SCD1 mRNA expression, AA+DPA showed a significant decrease in the mRNA expression of SCD1 again suggesting a dominant effect of AA. The most interesting observation was that treatment with EPA alone increased SCD1 mRNA, while AA treatment caused a decrease in SCD1 mRNA expression; however, a combination of EPA+AA showed no change in the mRNA expression of SCD1. These findings demonstrate a cancellation effect of a combination of AA+EPA. Mature adipocytes treated with AA+EPA showed a significantly higher accretion of AA, suggesting that AA is preferentially taken up or stored by adipocytes over EPA, which is responsible for the dominant effect of AA. A higher concentration of n-6 PUFA was reported in visceral and subcutaneous adipose tissue of morbidly obese patients that was associated with lower expression of SCD1 mRNA (29). Inhibition of SCD1 promotes inflammation, atherosclerosis, and pancreatic β-cell dysfunction in preclinical rodent models (30, 31), thus high concentrations of n-6 PUFA will suggest detrimental effects.

Overall, our findings have shown that long-chain n-3 PUFA and AA has different regulatory effects on adipogenic and lipogenic genes in adipocytes. We also confirm a dominant effect of AA when given in combination with LC n-3 PUFA on the metabolic regulation of adipocytes, which was due to higher accretion of AA over n-3 PUFA in mature adipocytes. The Western diet is high in AA, thus a preferential effect of AA may be the reason for detrimental health effects. Our findings reinforce the importance of increasing the consumption of n-3 PUFA to maintain the regulation of adipocytes metabolism.
